# An evolutionary algorithm for the discovery of porous organic cages†
†Electronic supplementary information (ESI) available: Further details about the fitness function calculation, setup of optimization runs, and the properties of the cages found in the final population for all the case studies. The structure files of the top five candidates in each run are also provided. See DOI: 10.1039/c8sc03560a


**DOI:** 10.1039/c8sc03560a

**Published:** 2018-09-11

**Authors:** Enrico Berardo, Lukas Turcani, Marcin Miklitz, Kim E. Jelfs

**Affiliations:** a Department of Chemistry , Imperial College London , South Kensington , London , SW7 2AZ , UK . Email: k.jelfs@imperial.ac.uk ; Tel: +44 (0)207 594 3438

## Abstract

The chemical and structural space of possible molecular materials is enormous, as they can, in principle, be built from any combination of organic building blocks. Here we have developed an evolutionary algorithm (EA) that can assist in the efficient exploration of chemical space for molecular materials, helping to guide synthesis to materials with promising applications. We demonstrate the utility of our EA to porous organic cages, predicting both promising targets and identifying the chemical features that emerge as important for a cage to be shape persistent or to adopt a particular cavity size. We identify that shape persistent cages require a low percentage of rotatable bonds in their precursors (<20%) and that the higher topicity building block in particular should use double bonds for rigidity. We can use the EA to explore what size ranges for precursors are required for achieving a given pore size in a cage and show that 16 Å pores, which are absent in the literature, should be synthetically achievable. Our EA implementation is adaptable and easily extendable, not only to target specific properties of porous organic cages, such as optimal encapsulants or molecular separation materials, but also to any easily calculable property of other molecular materials.

## Abstract 2

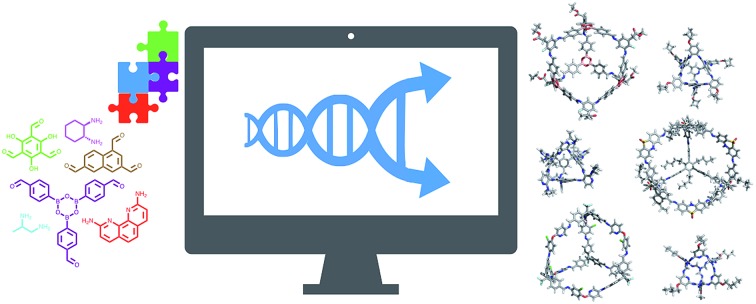
An evolutionary algorithm is developed and used to search for shape persistent porous organic cages.

## Introduction

Porous molecular materials are an emerging class of porous materials, which, unlike network solids such as zeolites, polymers and metal–organic frameworks (MOFs), lack extended chemical bonding and are instead built from discrete molecular units.^1^,^2^ Porosity in the solid-state can be achieved for porous molecular materials through either extrinsic porosity, where the molecule is unable to pack so as to fill all void space, or through intrinsic porosity, where the molecule itself has an internal cavity. Examples of the latter include molecular cages, which in addition to an internal cavity, have multiple entry and exit windows,^3^ belt-like molecules, such as cucurbiturils and cyclodextrin, or bowl-shaped molecules, such as calixarenes. Typically, in order to maximise the porosity in the solid-state, the relatively rare feature of ‘shape-persistency’ is being sought; the molecule must retain its intrinsic cavity in the absence of any stabilising solvent. Recent efforts have afforded record-breaking surface areas of 3786 m^2^ g^–1^ for a boronate cage, with a molecular diameter of ∼3 nm,^4^ and 3425 m^2^ g^–1^ for a triptycene-based building block with extrinsic porosity.^5^

Potential applications of porous molecular materials include as encapsulants,^6^ in catalysis,^7^ molecular separations,^8^–^11^ and as sensors.^12^,^13^ Most promising are applications in molecular separations, where some molecules have been found to be effective for gas separation,^11^ separation of aromatics,^9^ separation of alkanes/alkenes^8^,^14^ and chiral separations.^11^ Several of these applications occur in solution rather than in the solid-state, a unique possibility due to the molecular nature of the material. More recently, an intriguing new application has been reported – the use of molecular cages to form porous liquids.^15^

Porous molecular materials still only number in their hundreds, compared to, for example, the many hundreds of thousands of MOFs reported.^16^ Computation has been playing a significant role in the discovery of new porous molecular materials.^17^ Evans *et al.* screened the Cambridge Structural Database to identify possible porous molecular materials from previously reported crystal structures.^18^ Using machine learning, they identified that a large molecular surface area was a predictor of high crystal porosity. For molecular cages, one of the challenges of *a priori* prediction is the emergent behaviour in the reaction outcome from the molecular precursors. Simple changes to the precursors can double the mass of the product, changing its molecular topology and consequently its properties, for example losing any potential porosity through loss of shape persistency.^19^ We have previously outlined the 20 most likely topologies for molecular cages, split across four families, which are distinct based on the number of precursor reactive end groups.^20^ Furthermore, the potential for computational prediction of the reaction outcome by comparing the relative energy of the different assemblies^20^,^21^ or considering their reaction pathways has been shown.^22^ Recently, we tested this hypothesis on a larger scale in a combined robotic and computational screening, where 33 new porous organic cages were discovered.^23^ This demonstrated the value of computation; the topological outcome could be predicted where there were sufficiently large thermodynamic driving forces, and further, formation energies of the cages could be correlated with the likelihood that a molecular cage as successfully formed on the robotic platform. Calculations can therefore prevent synthetic effort being wasted on unpromising reactions, and also, through further computational property calculations, allow full scale-up and characterisation to only go ahead on the most promising materials.

At the solid-state level, it has been shown that the low energy polymorphs and therefore crystal packing of porous molecular materials can be predicted using crystal structure prediction techniques originally developed for pharmaceuticals.^24^–^27^ Most recently, this approach has been used to calculate energy–structure–function maps and thus guide the discovery of targeted properties in extrinsically porous triptycenes.^5^ For intrinsically porous molecular materials, there have been many computational studies demonstrating the possibility of predicting separation performance in both the crystalline and amorphous solid-state, through, for example, calculation of the pore network, sorption uptake and diffusion barriers for kinetic separations.^10^,^11^,^28^,^29^ In all cases, a consideration of the flexibility of the porous molecular host has been shown to be critical, particularly in understanding the sorption of guests that look too large to diffuse through the systems from inspection of static crystal structures alone.^30^ Recently, we have demonstrated the potential for rapid property screening of porous molecular materials through a molecular analysis of the molecules alone, without considering the bulk structure or influence of crystal packing.^31^ This approach allowed us to discover a promising previously reported material, noria, whose potential for Xe/Kr separation we then validated. In the area of metal–organic polyhedra, Hay *et al.* have developed the software HostDesigner, which designs molecular receptors for guests.^32^–^37^

The chemical and structural space of hypothetical porous molecular materials is enormous, given they are formed from building blocks of organic molecules. Most porous organic cage systems to date have been synthesised using dynamic covalent chemistry (DCC), where the reversible nature of the reaction allows error correction towards high symmetry, discrete structures. If we consider the most commonly used chemistry for cage synthesis, imine condensation, then inspection of only the online Reaxys database of previously reported molecules,^38^ finds on the order of 10^5^ total aldehyde and amine precursors. If you combine these into two-component cages in all possible topologies, then you already have 10^7^ potential host molecules to consider. For this reason, we have developed an evolutionary algorithm (EA) for the prediction of molecular materials, and demonstrate here its application to the discovery of porous molecular materials. EAs are inspired by biological evolution, where candidates are evaluated for their ‘fitness’, which determines their likelihood of proceeding to a subsequent generation. At each generation, several candidates will undergo random mutations and pairs of candidates will ‘reproduce’, with their chromosomes undergoing crossover. EAs, such as genetic algorithms, have been widely applied in chemistry, including for structure prediction^39^ or determination and in drug discovery.^40^ EAs efficiently search disparate regions of multidimensional phase space with multivariable optimisation and are particularly well suited to problems where a computationally cheap calculation can be used to determine a candidate's fitness, as can be the case for porous molecular materials.

Evolutionary methods have been developed both for experimental^41^–^43^ and computational^44^–^55^ materials discovery and optimization. To date, very few evolutionary approaches have been employed for the study of molecular materials, as this involves multiple obstacles, such as (i) the huge diversity in chemical bonds and flexibility in regards to electronic and structural properties, (ii) the need for a framework for the assembly and the correct generation of the molecular structure, and (iii) the lack of cheap and accurate descriptors that link structure with the potential properties of the final material in many cases.

In this work, we report on the extension of our previously reported software, the supramolecular toolkit (*stk*),^56^ which can assemble a variety of (supra)molecular materials, including porous cages, and then automate their property calculation. *stk* is written in python and makes use of utilities within the RDKit cheminformatics libraries.^57^ Within *stk*, each EA individual is defined as a Python object, which allows for the labelling and tracking of its compositional (*e.g.* precursors, atoms, bonds) and structural (*e.g.* cavity size, diameter, total energy) properties throughout the evolutionary run. Whilst here we develop the EA for the discovery of cages, it is easily extendable to other (supra)molecular materials in the future. We focus on the molecular prediction of cages, having previously demonstrated the utility of this approach for property prediction,^31^ and as this significantly decreases the calculation cost of the fitness function compared to solid-state calculations. After first showing that our approach can efficiently rediscover a previously reported cage, we then apply the software to two distinct case studies: (i) targeting elusive shape persistent cages and (ii) targeting a specific pore size, 16 Å, which has not been previously synthetically reported. This will show the utility of our software, which could easily be targeted at specific properties, such as encapsulation or molecular separation, in the future for porous molecular materials, or extended to the broader field of (supra)molecular materials.

## Workflow of the EA

**Fig. 1:**
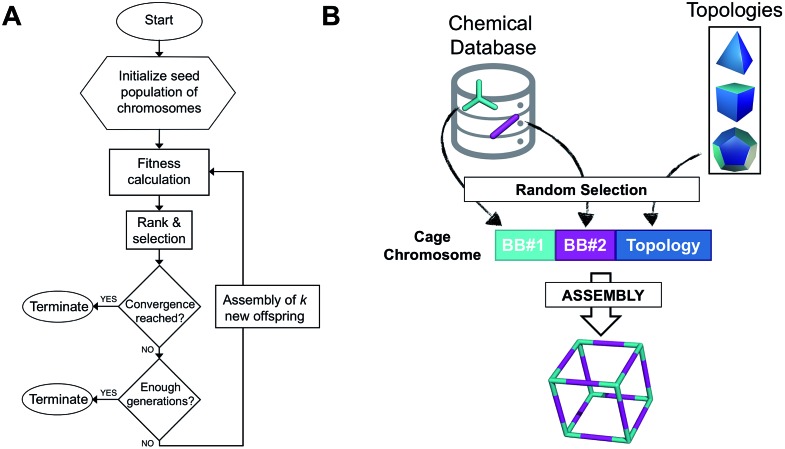
(A) Overall workflow of the EA for porous organic cages. *k* represents the number of offspring individuals that temporarily extend the population size at each generation. (B) Definition of the cage chromosome, which is composed of three genes, BB#1, BB#2 and a topology. Both BBs are randomly selected from a chemical database, whereas the topology is selected from a list of feasible topologies depending on the topicity of the BB. The cage is then assembled into a molecular cage structure.

The overall structure of our EA is shown in Fig. 1A. The seed population of starting porous organic cages is obtained by assembling molecular structures selected from a database of chemical precursors. The assembly of two-component organic cages is briefly described in the following section, but follows the procedure that we described for our supramolecular toolkit (*stk*),^56^ which assembles molecular materials, including porous organic cages. Here we describe the extension of *stk* to the exploration of chemical space using an EA. Each organic cage (or individual) chromosome can be uniquely defined by a set of three variables (or genes): two building blocks (BB) and a topology, as shown in Fig. 1B. The chromosome representation is of fixed length; this cannot be increased or decreased during the EA run.

**Fig. 2:**
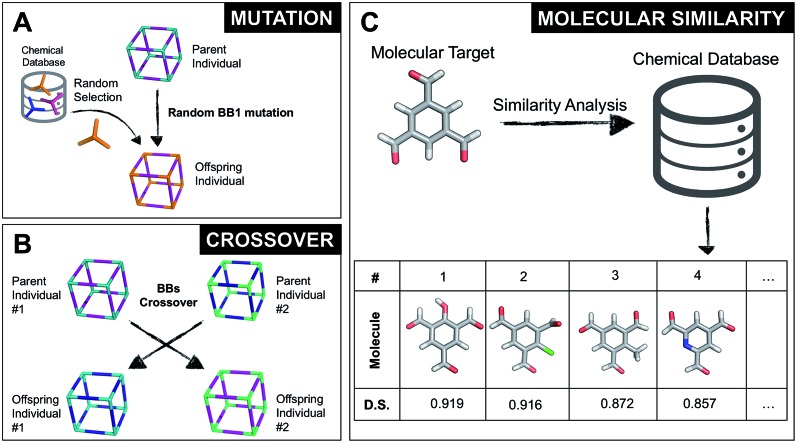
(A) Example of a mutation operation on a hypothetical two-component cubic parent cage, where BB1 is randomly replaced with a new tri-topic BB from the original chemical database. Alternative mutation types would involve exchange of the other BB or of the cage topology. (B) Example of a crossover operation on two hypothetical parent cages, where by mixing their genetic information (BBs), two new offspring are generated. Both (A) and (B) do not display real cages, but instead hypothetical assemblies where the chemical BB are simplified by sticks with the correct topicity. (C) Molecular similarity analysis for a specific molecule against the building blocks present in a chemical database. By employing Dice similarity, we select the most chemically similar molecules to our target molecular candidate. On the bottom we show the top 4 molecules with their corresponding ranking and Dice similarity (D. S.) value.

Once all the individuals in the initial population have been generated, each cage in the population has its fitness value evaluated (as described below). The individuals are ranked according to their fitness value and, depending on the specific selection algorithm, a set of parent structures is chosen. New offspring structures are generated starting from the selected parents by applying the genetic operations of crossover and mutation, shown in Fig. 2A and B. Both genetic operations modify the chromosome of a molecular cage by the substitution of its constituent genes. In the case of crossover, the genes of two parent cages are mixed to generate two new offspring, for example by switching BBs or topologies. For mutation, one of the existing genes is replaced by a new random one from a database of BBs or a list of topologies, respectively. The newly generated offspring structures have their fitness value calculated and they are used to replace the worst performing individuals from the current population, thus maintaining a constant population size. As shown in Fig. 1A, this cycle continues until a convergence criterium is met or the EA reaches the maximum number of allowed generations. Specific convergence criteria could be the appearance of a particular individual in the current population or that the top 5 cages remain unchanged for 20 generations, suggesting that the EA run has reached a plateau.

### Chemical databases

In our EA, the BBs selected for the generation of the initial seed population (and during the mutation step) come from a chemical database. Since this work focuses on the generation of porous organic imine cages, our initial database only contained aldehydes and amines and, in order to restrict the size of the chemical space to be explored, we only allowed for tri-topic aldehydes and di-topic amines. Our database was generated by mixing the free eMolecules database^58^ (containing around 18M molecules) and a selected portion of the proprietary Reaxys database^38^ (5 K di- and tri-topic aldehydes and 60 K di- and tri-topic amines). We removed all charged molecules, any molecule containing metals and any instances where the reactive amine was actually an amide, as this functionality will prevent imine condensation occuring. The final database contained 153 trialdehydes and 39 203 diamines. When considering two-component imine cages in one possible topology, the size of the chemical space amounts to around 6 M possible combinations. In the future, it would be possible to use custom databases or enumerate hypothetical libraries for a given search or material class, or to screen the library first to remove more chemically complex molecules that are the least promising for materials synthesis.

The 3D coordinates of each molecule within the chemical database were obtained starting from SMILES codes by using the ETKDG^59^ algorithm as implemented in RDKit, which allows for the efficient generation of reasonable conformations of small molecules. Differently from other recent applications of EAs for computational chemistry and materials discovery,^47^,^48^,^53^,^60^ we do not allow for the chemical modification of the original precursors during the EA search. In our implementation, the mutation step can only perform a substitution of one of its two building blocks of a cage with a completely new one from the chemical database. From an experimental point of view, we believe that this approach helps us to circumvent a common problem in EAs, where chemically infeasible candidate structures are generated that can not be synthetically accessed. BBs included from Reaxys have been previously synthetically reported and all cage structures built here can hypothetically be synthesised in a one-pot imine condensation reaction. Within this work, we do not investigate the synthetic accessibility of the final candidates, instead we allow any possible combination of the precursors available from the original database. However, synthetic accessibility can be addressed in the future by including synthetic rules or scoring in the EA's fitness function and refining the original chemical database or using a custom database.

By completely substituting one of the three genes of a chromosome, a mutation step strongly modifies the chemical/structural properties of a cage, possibly losing all the evolutionary advantage that has been developed up to that specific generation in a EA run. For this reason, we developed the similarity mutation, as shown in Fig. 2C, where the current gene (or building block) within a cage is replaced by the most similar BB from the database, as calculated with Dice similarity.^57^,^61^ Applying the similarity mutation multiple times to the same molecule yields the next most similar molecule in the list every time. For example, running the similarity mutation for the molecular target in Fig. 2C will lead to molecule #1 if run a single time, to molecule #2 the second time, and so on. In a usual EA run, we alternate between the random and similarity mutation, as we have found that this specific combination leads to a faster convergence of the run, allowing an efficient exploration of the overall chemical space.

## Cage assembly

**Fig. 3:**
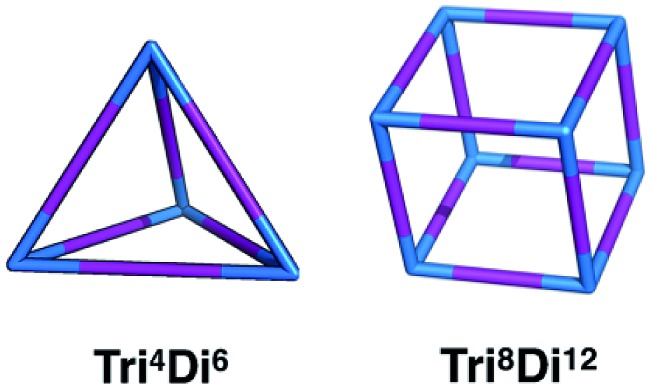
Schematics of the **Tri^4^Di^6^** and **Tri^8^Di^12^** topologies. **Tri**-topic and **di**-topic chemical precursors are represented by blue and purple sticks, respectively.

In this work, we investigate the family of molecular cages built by a combination of a tri-topic and di-topic BBs, where the BBs are combined in a 2 : 3 ratio.^20^ Within this family, for simplicity, we will only focus on cages with two different topologies, shown in Fig. 3, the **Tri^4^Di^6^** topology that relates to a tetrahedron and the **Tri^8^Di^12^** topology that relates to a cube. The superscripts from the nomenclature represent the number of precursor molecules with the specific topicity (**Tri** for **tri**-topic or **Di** for **di**-topic) included in the assembly. For example, the **Tri^4^Di^6^** molecule consists of four tri-topic and six di-topic building blocks. The nomenclature was recently suggested by us^20^ and we refer to that work for a more detailed explanation. By considering the two topologies defined above, the complete chemical space we are exploring increases to around 10^7^ possible molecular cages.

## Fitness function

Once each individual in the current population has been generated and geometry optimised, the evaluation of the fitness function allows for their ranking. The higher the fitness value of a candidate, the higher its rank within the current population. During an EA run, we should observe an increase in the maximum and average fitness values. We have developed a generalisable multi-objective fitness function, which can be used for a series of optimisation problems for porous organic cages, and is easily modifiable and extensible to include other terms and to other molecular materials. In our case, we do not necessarily seek pareto-optimal solutions, rather we use a scalarisation approach where the fitness function aims to allow compromise between the different objectives.

Our fitness function, shown in eqn (1), can be specified from a series of three parameters that we believe are important for the design of reasonable candidate porous organic cages. We chose each parameter after a detailed analysis of the structures of the porous organic cages recently synthesized in the literature.^1^,^2^,^20^,^62^ The parameters allow for the evaluation of the candidate's porosity, flexibility and degree of symmetry. Each parameter, which will be addressed in the next section, defines the penalty that is going to be applied to the fitness value of each candidate, the larger the penalty, the lower the final fitness function of the candidate. These various contributions can be combined inside the fitness function by using a series of coefficients (*a*, *b*, *c* in eqn (1)), so that the same general fitness function can be tuned for a variety of different applications or targets. More details on the specific implementation of the fitness function can be found in the ESI.† The fitness function is calculated as:

1 fitness = (*a*ΔPore + *b*ΔWindow + *c*Asymmetry)^–1^

where ΔPore refers to the difference in the measured pore size to the ideal pore size specified, and ΔWindow is the equivalent for the window size. The Asymmetry is an absolute value. These individual contributions are then normalised against the population average, as explained in further detail in the ESI.†

### Porosity

For porous molecular materials, the material's overall porosity can be linked to both intrinsic and extrinsic porosity. As already defined in the introduction, here we only investigate porous organic cages from a molecular perspective, not addressing the effects of packing multiple cages in the bulk. We have previously demonstrated that consideration of only the molecular structure can be effective even for screening for molecular separation applications of these materials.^31^ Here, we only consider the intrinsic porosity of a single cage and try to predict if the cage molecule will retain its internal cavity or tend to collapse. We perform a cavity analysis on the lowest energy conformer and compare the candidate's internal pore size to a pre-defined ideal pore size, the Pore parameter in eqn (1). The larger the deviation of the current pore size from the ideal size, the larger the applied penalty to the current candidate.

In a similar way, we can compare the window size of the candidate, calculated through the use of the software pywindow,^63^ against an ideal window size, the Window parameter in eqn (1). The cavity diameter is defined as two times the distance between the center of mass of the molecule and the closest atom (*i.e.* the diameter of largest sphere that can fit in the centre of the host molecule), whereas the diameter of a window is determined by the largest circle that can fit in the window.

### Symmetry

The vast majority of organic cages experimentally synthesised in the literature exhibit a high level of symmetry.^1^,^2^,^20^,^62^ We therefore try to evaluate how symmetric the lowest energy conformer extracted from the MD simulation is by comparing the size of all its chemically identical windows. The sum of all the windows' pair differences represents the asymmetry of the individual, Asymmetry parameter in eqn (1); more asymmetric cages are penalized to a greater extent and will have a lower final fitness value. A detailed explanation of this procedure can be found in the ESI.† In the case studies discussed within this work, we aim at minimizing the asymmetric penalty observed in cages. However, highly unsymmetrical assemblies could be employed in advanced chemical applications and thus the EA could be run so as to seek to maximise asymmetry of the cage molecule, which could be useful for applications such as porous liquids.^15^

The cage structures were assembled using our *stk* software, as previously described.^56^ In brief, a molecular cage is generated by placing the tri-topic BBs on the vertices of the topology, whereas the di-topic BBs are placed on the topology edges, which connect two adjacent vertices. Once the BBs are placed on the hypothetical topology, the relevant atom groups are then linked through imine bonds, which replace the existing functional groups of the original BB (*e.g.* aldehydes and amines). The newly formed bonds and the atoms directly linked to them are initially relaxed while constraining the remaining atoms within the molecular cage. Following this, the geometry of the whole molecular cage is optimised. Finally, we employ high-temperature molecular dynamics (MD) to probe the flexibility of the optimized individual and its tendency to retain its original cavity – also known as its “shape persistency”. In this step, for each candidate, we perform the MD simulation and extract a series of conformers along the trajectory. We then geometry optimize all the extracted conformers and select the lowest energy conformer, which represents the best guess of the experimental geometry upon desolvation (more information on this step can be found in the ESI†). In all stages of the geometry optimisation, we employ the OPLS3 force field,^64^ which we have previously shown effectively predicts the structure of flexible porous imine cages.^20^

## Results and discussion

In this section, we will first introduce the optimization of our EA, in which we define a smaller mock chemical space to run multiple EA setups and select the best performing one in the search of the global minimum. We then employ this setup for two different case studies, by using slightly different fitness function parameters in each case. We discuss the trends and insights that can be extracted from the EA runs and suggest how this approach could be used to accelerate materials discovery for porous organic cages.

### EA optimization: **CC3** rediscovery

In our current implementation of the EA, the user can choose between different types of functions for the initial population generation or for the genetic operations of selection, crossover and mutation. For example, when creating a new population of individuals, the user can decide to randomly sample BBs from the chemical database to generate the required number of cages. Alternatively, the user can employ a diverse initialization, where a random selection of BBs from the database is alternated with the selection of dissimilar BBs (calculated *via* Dice similarity), assuring that different areas of the chemical space are explored. Similarly, the user can choose among five different selection functions for selecting molecules for the next generation, such as only selecting the fittest candidates at each iteration, or using roulette wheel (with or without elitism) or universal stochastic sampling.^65^,^66^ Details about all the possible functions can be found in the *stk* documentation.^67^

When facing this excess of functions and options, it can be difficult to know which settings, or combinations of settings, will lead the EA to have better or worse performance. To address this question, we ran the EA with 360 different input files, where each input file contained a distinct combination of functions available within our EA implementation. For each input file, 2500 individual runs of the EA were completed. We have run each setup multiple times as EAs are stochastic algorithms and the generation by which the global minimum is found is dependent on the initial population. Since the initial population and mutation operations are random – the generation by which the global minimum is found is given by a distribution of values. A run was considered complete whenever the global minimum structure was obtained or whenever 100 generations were reached. Each run employed a fitness function developed for the re-discovery of Covalent Cage 3 (**CC3**),^68^ a well-known example of a highly symmetric **Tri^4^Di^6^** imine cage, with a pore size of 5.72 Å and average window sizes of 3.91 Å when OPLS3 optimized.

To allow for the consecutive execution of 900 000 EA runs (360 × 2500), we defined a smaller chemical space, for which we could pre-generate and optimize all the possible molecular cages. The fitness value of each individual was then pre-calculated with the fitness function targeting the **CC3** cage (pore size and window size used in eqn (1)). This means that for each EA run, the entire search space was loaded into memory and no calculations needed to be performed. Using this setup, 100 EA generations could be completed in approximately 1 s. It also meant that the individual with the largest fitness value, the global minimum **CC3** could be found ahead of time. The selected mock chemical space contained 142 trialdehyde BBs and 350 diamine BBs selected randomly from the initial chemical database, as well as the BBs for **CC3**. For this mock chemical space, we assembled, optimized and calculated the properties of all the possible **Tri^4^Di^6^** cages, which amounted to a total of 49 700 individuals.

In each EA run, we explored an initial population of 25 individuals, allowing 20 possible crossover operations and 5 mutations per generation for up to 100 generations. This means that in a EA run which successfully reaches 100 generations, 2500 possible individuals are explored, corresponding to only the 5% of the total chemical space. As shown in Table S2,† the different EA setups have a probability of finding the **CC3** cage that ranges between 0 and 59% of the times (over the 2500 runs). The best performing setups show a huge improvement when the EA is compared to randomly picking BBs from the chemical database, which only gives a probability of 5% of finding the global minimum. Among the best performing setups, we chose the one including the diverse initial population as we believe that this type of initialization function will perform even better with the large chemical space used for the following case studies. All the functions used for the EA setup can be found in the ESI.†

The fitness function for this investigation employed the coefficients *a* = 10, *b* = 10, *c* = 1 from eqn (1), where we defined ideal pore and window diameters of 5.72 and 3.91 Å, respectively. We wanted to put a strong emphasis on the pore and window sizes in order for the global minimum to precisely match the properties of the OPLS3 optimized **CC3** cage. We also gave some importance to the level of symmetry of the cage.

**Fig. 4:**
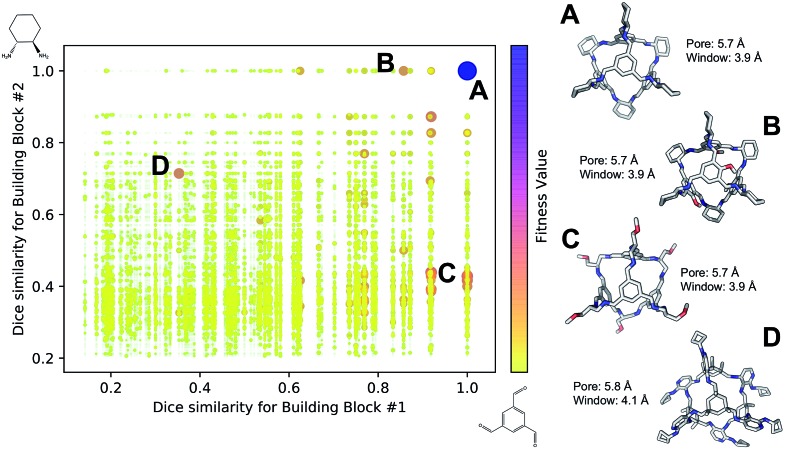
Fitness value distribution of the 49 700 **Tri^4^Di^6^** cages obtained for **CC3** re-discovery runs plotted against the molecular similarity of the cage BBs to **CC3** BBs. Cages with higher fitness values are represented with darker and larger dots (purple), whereas low fitness value is defined by small yellow dots. **CC3** is shown as (A) on the right of the figure, along with three examples of cage individuals with medium (B–D). For the molecular cages, C, N, O and H atoms are displayed by light gray, blue, red and white, respectively. Non-polar H atoms are hidden.

Fig. 4 shows the distribution of the fitness value for the 49 700 molecular cages obtained, plotted as a function of molecular similarity with the BBs used for **CC3** (triformylbenzene and cyclohexane diamine). Molecular similarity is calculated by means of Dice similarity through the use of Morgan fingerprints with a standard radius of 1 within RDKit.^57^,^69^ The plot clearly shows that the individual with the highest fitness value is **CC3**, located on the top-right point of the plot (Fig. 4A). No other solution exhibits such a high fitness value, confirming that the fitness value was tightly linked to the global minimum structure (**CC3**). However, a few other individuals have medium fitness values (dark-orange, larger dots). For most of those cases, at least one of the BBs shows high Dice similarity with the **CC3** BBs (Fig. 4B and C). Both B and C cages have very similar (equivalent to the first decimal place) pore and window sizes compared to **CC3**, but show overall lower fitness value due to a decreased level of symmetry. Cage B is obtained by mixing a cyclohexane diamine and a substituted (–OH) triformylbenzene, whereas for cage C, triformylbenzene is mixed with a different diamine. Some interesting cages still exhibit a medium fitness value even if their precursors are very different to the ones employed for **CC3** (Fig. 4D). In this case, one BB is highly functionalized compared to the **CC3** diamine, but this functionalisation is external to the cage–core, and thus barely effects the window and size parameters that we used for the fitness function. The use of chemically different BBs does however lead to a more flexible cage with a slightly larger pore, window sizes and overall lower fitness value.

This example highlighted the efficiency of our EA implementation. The EA search is clearly more efficient than a brute force approach, as only a small fraction of all the possible solutions is selected for computational investigation. However, we stress that although EAs are among the best performing methodologies to probe these vast spaces, the restricted length of each EA run and its stochastic character, do not allow for a comprehensive exploration of the solution space. Solutions will generally be high fitness local minima from very complex multidimensional surfaces, and are very unlikely to be the global minimum.^51^ By aiming to accelerate the discovery of new materials, we are not only interested in the global minimum for a fitness function, but instead any high-fitness solution is a candidate for experimental realisation. We show in the next two case studies how high-fitness candidates, which do not necessarily represent the global minimum, can be used to extrapolate design patterns for new materials.

### Case study 1: target shape persistency

As already discussed in the introduction, finding porous organic cages that are ‘shape persistent’ is a challenging task. Shape persistency, which is the tendency of a porous cage to retain its original cavity upon desolvation, strongly correlates with the rigidity of the assembly. From the analysis of the cages generated in the previous section, we calculated that only approximately 2% of the total cages were shape persistent (further details regarding how we assess shape persistency are in the ESI†). This scarcity of shape persistent cages is a problem, a research team can spend more than 1 year synthesizing and characterizing a molecular cage, only to then discover that the assembly is not shape persistent,^19^ making the material discovery process inefficient. With this case study, we show how the EA can be employed for a very quick screening of potential candidates, by directly assessing their shape persistent character. We explore our full chemical space to investigate the shape persistent character of porous organic cages with two possible topologies, **Tri^4^Di^6^** and **Tri^8^Di^12^** (approximately 12M possible combinations). Here we use a fitness function with the coefficients *a* = 5, *b* = 1, *c* = 10, where in this case the strongest evolutionary pressure comes from the asymmetry parameter, while the pore and window size (ideal diameter of 5 Å) play a minor role.

All the other parameters (such as selection functions and population size) are equivalent to the ones used for the **CC3** rediscovery, with the only difference being the convergence criteria. An EA run was considered to have reached convergence whenever its top 5 candidates remained unchanged for more than 20 generations. This setting allows us to avoid the continuation of a EA run whenever a stable plateau is observed and the probability of finding better performing candidates is very low. Due to the stochastic nature of EAs, we ran this setup 3 different times.

**Fig. 5:**
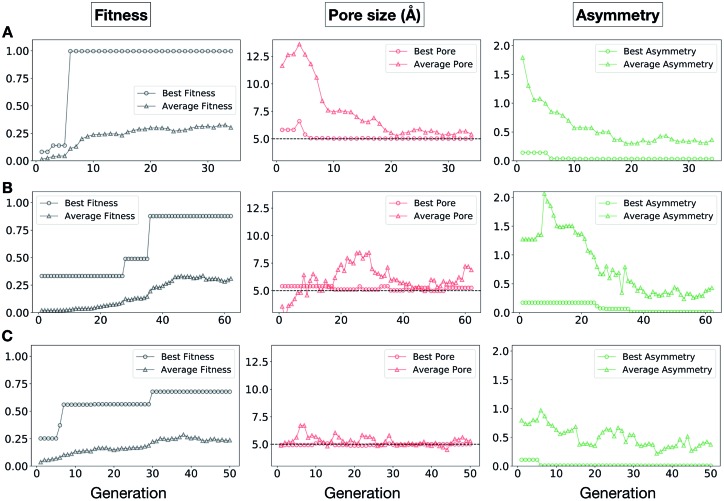
Evolutionary progress plot for three different EA runs (A–C), where shape persistency was targeted in **Tri^4^Di^6^** and **Tri^8^Di^12^** cages. For all plots the *x*-axis is the number of generations and the *y*-axis is as labelled at the top. The plots on the left (black) show the behaviour of the average (triangles) and best (circles) fitness value for the individuals for each generation. The middle plots (red) display the behaviour of the best (circles) and average (triangles) pore size for the individuals for each generation. The black dashed line defines the ideal pore size (5.0 Å). The right plots (green) display the behaviour of the best (circles) and average (triangles) asymmetry for the individuals for each generation. Lower values of asymmetry represent more symmetric structures.

In Fig. 5, we show the evolutionary progress plots for the three different EA runs performed for this case study. All of the runs converged in less than 100 generations (34, 62 and 50 generations for A, B and C, respectively). The total fitness plots (black) show that the average fitness value of the population, in general, increases as a function of the generation number. The best candidates within the population consistently display a much higher fitness value compared to the average. The middle plots (red) show that the best individuals quickly reach the target value of 5.0 Å, even if the pore size penalty is much weaker compared to the asymmetry penalty. This likely reflects the abundance of cages with suitable pore sizes, *versus* those with low asymmetry values. The weak penalty for pores that are not at the target value is the likely reason why a larger variation is observed in the average pore diameters compared to average asymmetry between generations, as the pore size does not significantly affect the fitness value. The right plots (green) show that the asymmetry parameter converges steadily towards 0 (when all the cage windows are equivalent in size). Both the asymmetry of the best individual and of the population average are very low at the end of the EA runs.

**Fig. 6:**
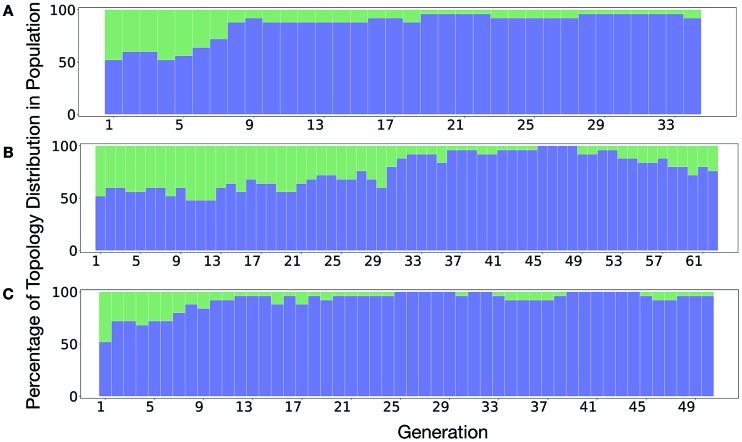
Percentage of different topologies observed during the different EA runs (A–C). The **Tri^4^Di^6^** and **Tri^8^Di^12^** topological contributions are represented as blue and green bars, respectively.

It is of interest whether a certain topology dominates when targeting a specific cage property. Fig. 6 shows the percentage of the two topologies in the populations for the three different runs. All three runs show a strong preference for the **Tri^4^Di^6^** topology, suggesting that this topology offers better control of the asymmetry when dealing with smaller cages (pore diameter of 4–11 Å), and thus offering better shape persistency. A non-negligible percentage of **Tri^8^Di^12^** is only observed in run B, where around 20% of the individuals in the final population possess the **Tri^8^Di^12^** topology.

**Fig. 7:**
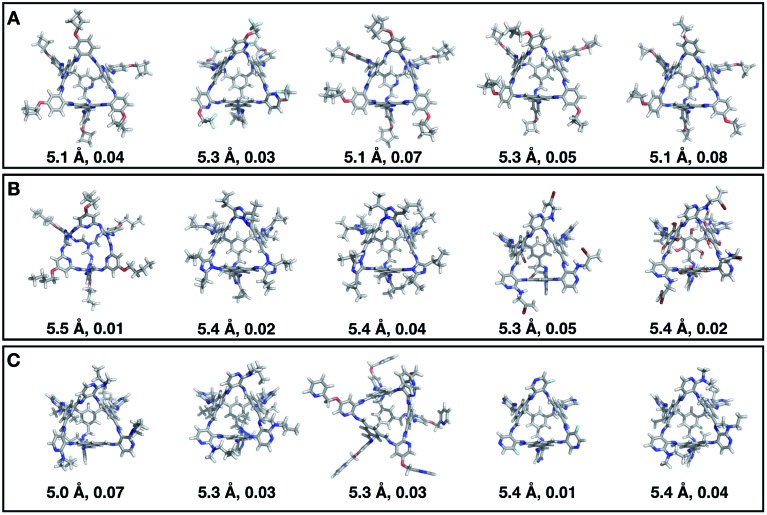
Top five candidates found within the last generation of each of the three EA runs for case study 1, where we targeted shape persistency in individuals with high symmetry and pore diameters of approximately 5.0 Å. The cages are ordered from left to right in decreasing rank number (#1 is on the left) and for each individual we provide the pore diameter and asymmetry value. C, O, N, F and H atoms are shown in grey, red, blue, light blue and white respectively.

Fig. 7 shows the top 5 cages obtained for each EA run with their corresponding pore diameter and asymmetry value. In the ESI† we provide the properties of the individuals selected for the last EA generation, and the structures of the top five for each run. The three different EA runs converged towards three different local minima, where for each run, multiple interesting BBs and therefore cages were explored. From Fig. 7, it can be seen that the best performing individuals are characterized by a combination of good pore size and a symmetric core. For all three runs, the top five cages are **Tri^4^Di^6^** individuals with pore diameters that fall within 0.5 Å of the target pore size of 5.0 Å. All cages share a very similar shape to that of **CC3**, typically with some external functionalisation. Some of the external functionalisation or heteroatom substitution on the rings may increase the complexity of the synthesis, however, these hypothetical cages could be simplified prior to any synthesis attempts. In this instance, the external functionalisation has limited or no influence on the fitness function, as this only depends on the pore size, window size and window symmetry. We only observed **Tri^8^Di^12^** individuals for two of the runs, at rank #16 and #19 within the runs (total of 24 candidates in a generation). This suggests that **Tri^4^Di^6^** cages are preferred when looking for rigid cages with smaller (5 Å) pore diameters.

**Fig. 8:**
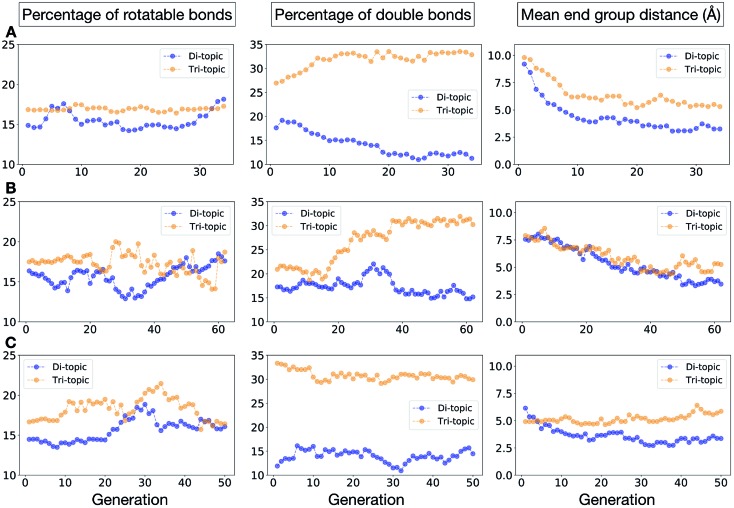
Analysis of the average molecular properties of the di-topic and tri-topic BBs for the individuals in each population of case study 1. For each EA run, we investigated the change in the percentage of rotatable bonds (left), percentage of double bonds (middle) and mean distance between reactive end groups (right). Di-topic BBs are represented by blue markers, whereas tri-topic BBs by orange markers.

To conclude this case study, we analysed the emerging patterns for the molecular properties of the BBs for the individuals in the population. In Fig. 8, we plot the behaviour of the percentage of rotatable bonds, double bonds, and the size of the BBs. The percentage of rotatable bonds and double bonds of a molecule was calculated as the ratio of the bonds of interest over the total number of bonds in the molecule. In all three runs the percentage of rotatable bonds converges towards ∼17% for both di-topic and tri-topic BBs, although there is considerable noise in this value. All three runs display a difference in the percentage of double bonds between the di-topic and tri-topic precursors, with the number for the tri-topic molecules being significantly larger (about 30% compared to 15%). The difference in the percentage of double bonds between the di-topic and tri-topic BBs highlights the more important role that double bonds have on tri-topic precursors in imparting rigidity, and thus shape persistence, to the cage. The mean distance between the reactive end groups (*i.e.* amine nitrogen to amine nitrogen distance in a diamine and average aldehyde carbon to aldehyde carbon distance in a trialdehyde) is shown on the right-hand side of Fig. 8. This measure defines the approximate size of the BB and gives us an idea of how the size of the precursors is progressing as the EA moves towards the best solutions. For the di-topic BBs, the distance converges on a value of ∼3.5 Å, which is the typical distance between nitrogens in the 1,2-diamines that dominate in the runs and in the reported syntheses of **Tri^4^Di^6^** cages of this size in the literature. The mean distance for the tri-topic BBs is larger, at ∼5 Å.

### Case study 2: target pore size

**Fig. 9:**
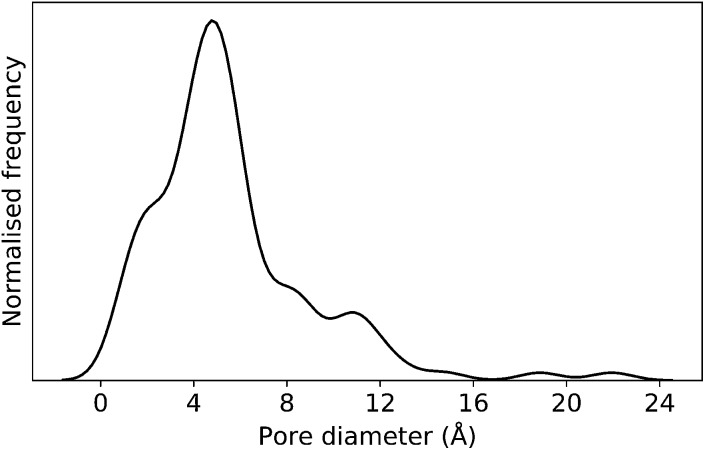
Distribution of pore diameters for 116 porous organic cages previously experimentally reported in the literature. There is a general absence of molecules with a pore diameter of 16 Å, or larger.

In this case study, we explore our full chemical space with the EA to find **Tri^4^Di^6^** porous organic cages with a cavity diameter of 16 Å. We have selected this cavity size as we found a clear absence of this pore size in the previously reported experimental pore sizes of 116 cages from the literature, as shown in Fig. 9.^2^,^70^,^71^ In part, this absence is likely related to the limited number of larger pores in general – once you are targeting pores of a larger size, it becomes increasingly likely that your cage will collapse. The largest reported cage is that from Zhang *et al.*, which we calculate to have an internal spherical cavity of 21.9 Å in diameter.^4^ We carried out a similar analysis of cavity size on the shape persistent organic cages from the mock space generated in the **CC3** rediscovery section (a total of 5772 cages), as shown in Fig. S1.† Similarly to Fig. 9, there is large peak in the range between 0 – 6 Å, with no cages showing pore sizes larger than 24 Å. This suggests that synthesising organic cages with shape persistent pores above 24 Å would be very challenging, and would require novel precursor design. The fitness function used for case study 2 employed the coefficients *a* = 10, *b* = 0, *c* = 5, where the ideal pore diameters matched 16.0 Å. This specific set of parameters was chosen in order to give a clear advantage to the pore size diameter, making it the driving force for the evolutionary pressure. All the other setup details are equivalent to the ones from the previous case study.

**Fig. 10:**
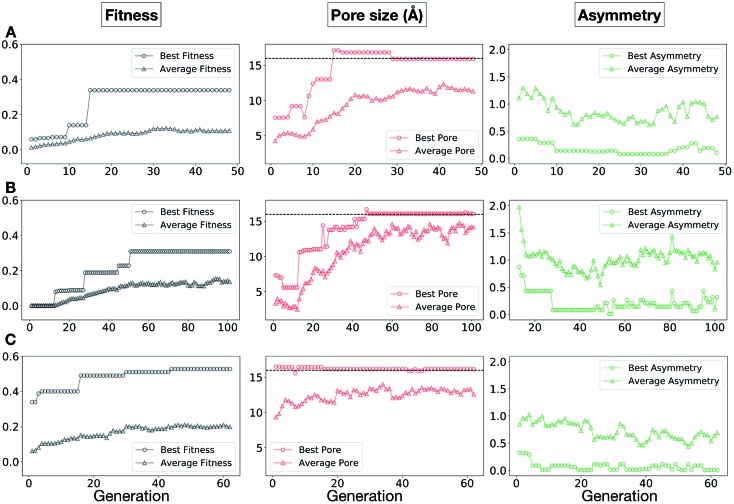
Evolutionary progress plot for three different EA runs, (A–C), where we targeted **Tri^4^Di^6^** porous organic cages with a cavity of 16.0 Å. The plots on the left (black) show the behaviour of the average (triangles) and best (circles) normalized fitness value for the individuals for each generation. The middle plots (red lines) display the behaviour of the best (circles) and average (triangles) pore size for the individuals for each generation. The black dashed line defines the ideal pore size (16.0 Å). The right plots (green lines) display the behaviour of the best (circles) and average (triangles) asymmetry for the individuals for each generation.

The plots in Fig. 10 show that for runs A and C, the EA converged in less than 100 generations (48 and 62 generations, respectively). The middle plots (red) indicate that in the first two cases, a constant increase is observed in the average value of the pore size within the population and the best cage found during the run has a pore size of approximately 16 Å, as requested. For run C, the initial population had much larger pore sizes on average and as a result the generation number does not lead to significant changes in the pore size. The plots on the right (green) correspond to the behaviour of the asymmetry parameter. While generation by generation the value of this property is rather random, there is a clear downward trend (towards more symmetric cages) over the course of the EA in all three cases, reflecting the evolutionary pressure placed on this parameter. We note that there is some conflict between attempting to generate cages with a large pore size and minimizing the asymmetry. As the asymmetry factor is calculated as the sum of the differences between all the topologically equivalent windows within a cage, it is clear that as the size of the cage increases (larger pore diameter and window diameter are correlated), we would expect the asymmetry factor to increase as well. This means that as the individuals are approaching the 16.0 Å target diameter, finding cages with lower asymmetry becomes progressively more challenging.

**Fig. 11:**
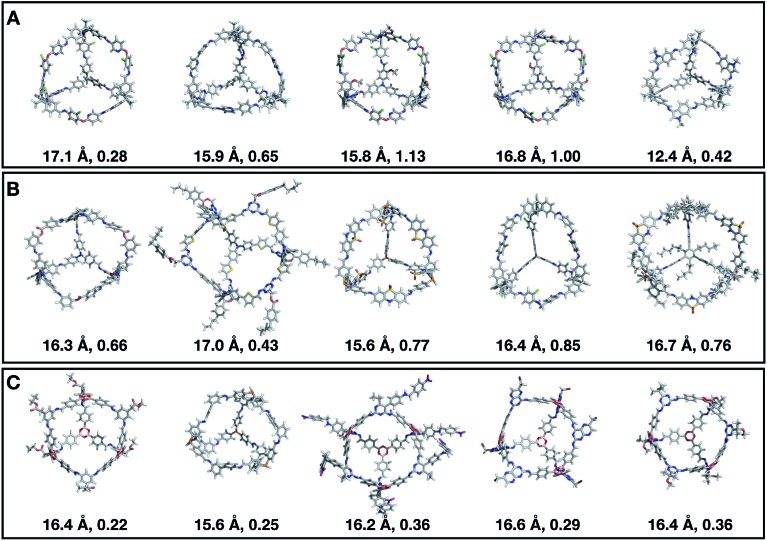
Top five **Tri^4^Di^6^** candidates found within the last generation of each of the three EA runs (A–C) of case study 2, where we targeted the best individuals with pore diameters of 16 Å. The cages are ordered from left to right in decreasing rank number (#1 is on the left). Underneath each cage we provide their pore diameter in Å and the asymmetry value. C, O, N, H, B, S, F, and Cl atoms are represented as grey, red, blue, white, pink, yellow, green and cyan sticks, respectively.

**Fig. 12:**
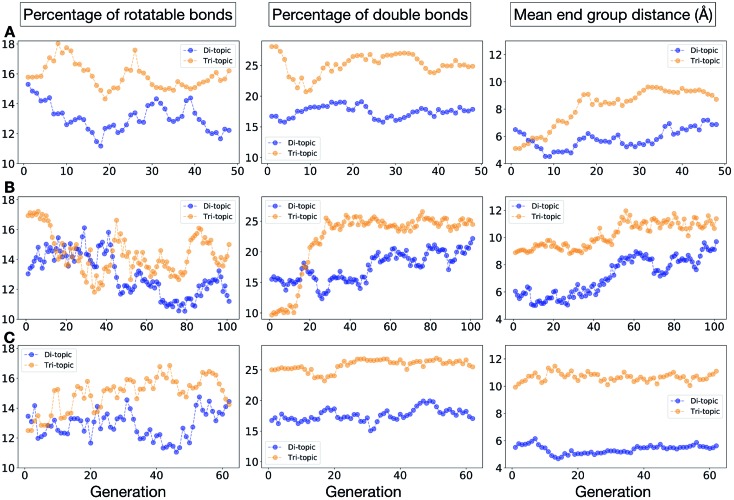
Analysis of the average molecular properties of the di-topic and tri-topic BBs for the individuals in each population of case study 2. For each EA run, we investigated the change in the percentage of rotatable bonds (left), percentage of double bonds (middle) and mean distance between reactive end groups (right). Di-topic BBs are represented by blue markers, whereas tri-topic BBs by orange markers.

Fig. 11 shows the top five cages obtained for each EA run, with their corresponding pore diameters and asymmetry values. In the ESI,† we provide properties of the individuals in the last EA generation and structures of the top 5 candidates in each run. Run C found a series of minima containing a boronate tri-aldehyde BB, whereas both run A and B share many cage individuals containing a triphenylamine. For the most part, however, the individuals are constructed from different precursors, with the most common feature being their similar size. As with the previous case study, identified candidates could be simplified, for example removing unnecessary functionalisation to make synthetic realisation more facile. Fig. 12 analyses further the emerging features of the BBs over the runs. The percentage of rotatable bonds for both di-topic and tri-topic BBs is very similar to that of the previous case study that sought smaller shape persistent cages, although slightly smaller at ∼14% compared to ∼17%, but again there is a lot of noise in these values suggesting this is not the sole critical consideration. Similarly, the percentage of double bonds in the BBs has decreased slightly for the tri-topic BBs by about 5%, and increased very slightly for the di-topic BB, although the values vary across the runs (∼17–22%), suggesting no strong changes in these characteristics of the BBs for targeting shape persistency in larger rather than small pores.

As would be expected, the size of the BBs required to target a large pore (16 Å) shape persistent porous organic cage has considerably increased compared to the previous case study of a small pore cage (5 Å). The mean distance between end groups here is 9–11 Å for tri-topic BBs and 6–9 Å for di-topic BBs (compared to ∼5 and 3.5 Å in case study 1). It is also interesting to see that each of the runs evolves towards slightly different size BBs (hence the range of values quoted), suggesting that there are multiple different solutions for shape persistent cages of 16 Å from the database of BBs we explored. It is likely this is due to the interplay between the size of the two BBs, where a larger di-topic BB could compensate for a small tri-topic BB than an alternative solution. We believe that this is an encouraging sign that porous organic cages with an internal cavity diameter of 16 Å are synthetically achievable, despite the absence of synthetic reports to date.

## Conclusions

We have developed an evolutionary algorithm for the prediction of molecular materials with desirable properties. Here, we have applied this to the field of porous molecular cages, where the enormous chemical and structural space of possible molecules makes an efficient sampling crucial. In our flexible implementation, a crossover operation is performed by switching the BBs of two candidate molecules or their topology, and a mutation operation is performed by switching in a new BB from the library. We found that our exploration of hypothetical cages was far more efficient when we included the possibility for mutation to a molecularly similar BB, rather than losing a large portion of the chemical information of a cage in a single mutation step. After demonstrating the effectiveness of our software for the rediscovery of a known cage, **CC3**, we then carried out two case studies to demonstrate our EA's utility.

**Fig. 13:**
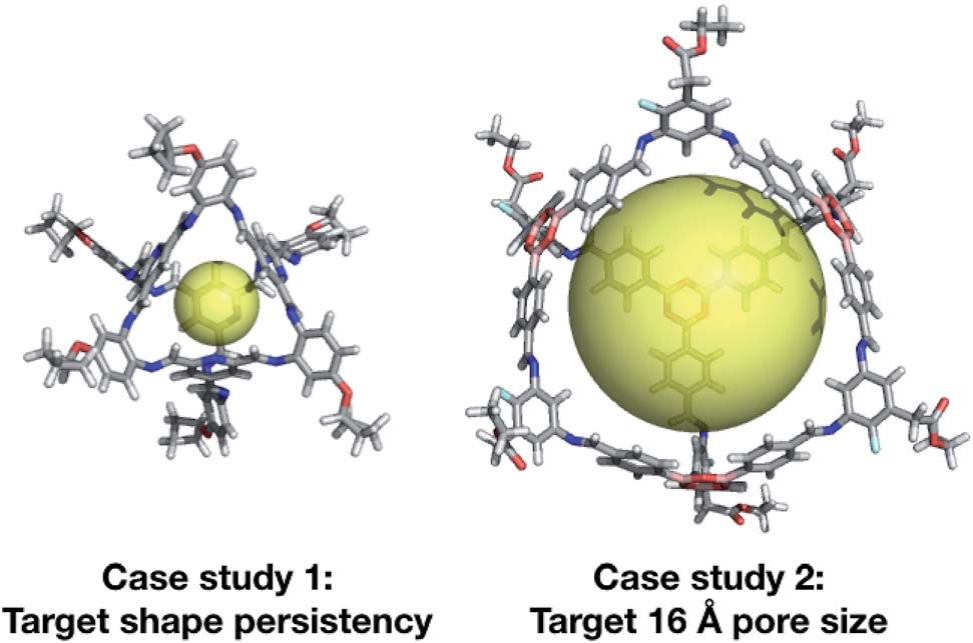
Top cage individuals from the two different case studies. Both cages are displayed with a transparent yellow sphere, which approximately defines the size of its internal cavity (∼5 and ∼16 Å). The external functionalisation of the diamines is not essential for the high scoring of the molecules and could be removed for ease of synthetic realisation.

Our two case studies allowed us to not only generate both specific targets, but further to explore the emerging trends of candidates with the desired properties, so we can identify key chemical features for a given property. We have identified that shape persistent porous organic cages require BBs with a very low percentage of rotatable bonds (<20%), and high percentages of double bonds, with tri-topic BBs in **Tri^4^Di^6^** cages requiring a higher percentage (25–30%) than the di-topic BBs (15–20%). The best candidate from each of the two case studies is shown in Fig. 13. Although there is no guarantee that the predicted compounds can be synthetically realised, we believe that our approach can be used to extract interesting structural motives from the high-fitness individuals of the last generations, which are worthwhile to study experimentally. Similarly, the targets could be simplified, for instance removing unnecessary external functionalisation to increase the ease of synthesis. In our first case study, we targeted cages that were shape persistent, with a small intrinsic internal cavity. The evolutionary pressure for symmetric small pore cages meant that **Tri^4^Di^6^** topology cages were strongly selected for, and all bore a visual similarity to the **CC3** series of cages, and were built using di-topic BBs with the typical nitrogen–nitrogen separation of a 1,2-diamine.

In our second case study, we targeted a cage with a cavity of 16 Å diameter, as we found that this was a cavity size absent in previously synthesised cage molecules. The best candidates among **Tri^4^Di^6^** cages typically chose a large tritopic building block, for instance the boronate structure shown in Fig. 13. In general, the high-scoring cages contained large building blocks with mean distances between end groups of 9–11 Å for tri-topic BBs and 6–9 Å for di-topic BBs. The range of different solutions found in different runs suggests that there are multiple different solutions for a shape persistent 16 Å pore, and that this should encourage attempts to experimentally explore this target. For these case studies and for future use of our EA, narrowing down the size range and other properties of BBs required to synthesise molecular materials with a given property provides the opportunity for online libraries of BBs to be data-mined for such criteria, or for such BBs to be designed.

The evolutionary algorithm we have included into our supramolecular toolkit, *stk*, is designed to be flexible. The fitness function can be adapted for new design challenges by adding additional parameters. For porous cages, the next obvious step would be to use our EA for targeting specific properties, such as encapsulation of a particular guest, by, for example, seeking to maximise the host–guest binding energy, or improving the separation performance by optimising window size or the diffusion barrier. If a given precursor is known to have a desirable property, such as fluorescence, which could be used for sensing, then the EA could be used to find a BB partner that forms a cage molecule of desired structure. Any designed molecules could also be used as a first step for crystal structure prediction, in order to produce optimal packing and properties in the solid-state. The EA can easily be applied to alternative molecular materials – indeed *stk* can already automatically assemble linear polymers, covalent organic frameworks (COFs), and small molecules built from multiple BBs. These molecular materials could then be explored with the EA if a fitness function for them is defined.

In the future, we also wish to extend the EA to make the predictions more facile to synthetically realise. Already, by using a database of known BBs and combining them in what would equate experimentally to a one-pot synthesis, we increase the likelihood of the candidates being synthetically achievable. Of course, much of the Reaxys and eMolecules databases feature molecules of high chemical complexity, this could be countered by including a score of synthetic accessibility^72^ or a synthetic chemist's scoring^73^ as part of the fitness function, or by developing a target library specifically designed for the synthesis of a given class of materials. As artificial intelligence is set to revolutionise materials discovery, there is the potential to couple our EA with machine learning (ML),^74^ through either using the EA to provide training data for a ML algorithm or to maximise both the computational efficiency of a property calculation, whilst using the EA to effectively sample the enormous chemical and structural space of molecular materials.

## Conflicts of interest

There are no conflicts to declare.

## Supplementary Material

Supplementary information

Supplementary information
