# Supplementary material for: An evolutionary algorithm for the discovery of porous organic cages
Source: Chem Sci. 2018 Sep 11;9(45):8513–27. doi: 10.1039/c8sc03560a (PMC6251339; doi:10.1039/c8sc03560a)
Supplement: Supplementary file 2 [file SC-009-C8SC03560A-s002.pdf]

Electronic Supplementary Material (ESI) for Chemical Science.
This journal is © The Royal Society of Chemistry 2018

Electronic Supplementary Information for An Evolutionary Algorithm for the Discovery of Porous Organic Cages

Enrico Berardo, Lukas Turcani, Marcin Miklitz, and Kim E. Jelfs*

Department of Chemistry, Imperial College London, South Kensington, London, SW7 2AZ

E-mail: k.jelfs@imperial.ac.uk

Phone: +44 (0)20759 43438

Cage structure optimisation

One assembled, a high temperature MD run is performed to search for low energy conformations. Each cage undergoes an MD simulation for 2 ns after a 100 ps equilibration, with a timestep of 1 fs and at a temperature of 700 K. Every 40 ps, a structure is sampled and geometry optimised at OPLS3 level. The optimization process is divided in two steps. First, in order to decrease the total strain of the new assembly, only the newly created bonds are relaxed, keeping the bonds of the precursors fixed; in a second step, a full geometry optimization of the whole molecule is performed. The lowest energy conformation sampled is then used for further analysis. The convergence criteria for all geometry optimizations corresponds to a maximum of 2500 iterations and a gradient of 0.05.

Fitness function

As stated in the main text, the fitness value of a cage is given by:

$$fitness = (a\Delta\mathbf{Pore} + b\Delta\mathbf{Window} + c\mathbf{Asymmetry})^{-1} \tag{1}$$

where Δ**Pore** refers to the difference in the measured pore size to the ideal pore size specified, and Δ**Window** is the equivalent for the window size. The **Asymmetry** is an absolute value. Further, each component Δ**Pore**, Δ**Window** or **Asymmetry** is given by:

$$\mathbf{Comp} = \frac{\mathbf{Comp_{raw}}}{<\mathbf{Comp_{raw}}>} \tag{2}$$

where **Comp** represents one of the components in equation 1, $\mathbf{Comp_{raw}}$ represents the value of that component for the particular cage and $<\mathbf{Comp_{raw}}>$ represent the average $\mathbf{Comp_{raw}}$ among all the individuals in the population for a given generation. The formulation means that the $\mathbf{Comp_{raw}}$ is constant for a given cage, but **Comp** will vary depending on the other cages found in the generation. The normalization procedure involving division by

the average component value in each generation is necessary because the final fitness value involves a combination of components, which without scaling would have vastly different orders of magnitude. By dividing each value with the average in each generation, the quantity describes how much better or worse than the average a given **Comp** value is.

EA setup optimization

Each setup optimization run is defined by 4 numbers, "0000", where each number describes the ID of one of the EA operations employed in the following order, *Initialization Function*, *Generational Selection Function*, *Crossover Selection Function*, *Mutation Selection Function.* In this setup optimization, the choice of mutation type (*i.e. Similarity BB Mutation* or *Random BB Mutation*) was not tested, as preliminary results showed that including all the possible mutation functions led to a significantly improved performance. The difference between *Random Mutation* and *Similarity Mutation* is that, in the first case, one of the individual chromosomes (BBs) is replaced by a new random precursor coming from the chemical database to build a new individual. In the second case, the mutation function replaces the selected BB with a similar one from the chemical database. Similarity is measured by using the Dice similarity algorithm, which relies on the use of Morgan Fingerprints. The first time a BB is mutated, the most similar one is selected from the chemical database, the second time the next most similar precursor is chosen and so on.

Table S2 displays the results of the setup optimization run, done on the small chemical space (49,700 possible cages), where the ID of each setup can be referenced back to Table S1. The most efficient setups reach efficiencies higher than 50%, but there are also specific setups (**1111** and **0323**) for which no **CC3** was discovered among the 2500 EA runs. The top performing setups prove the advantage of using a EA compared to a brute force exploration of the chemical space, where the probability of finding one specific candidate over 49,700 possible solutions corresponds to 5%. From the top performing setups, we decided to select the setup where the *Diverse Initialization Function* was employed (**1433**), as we believed

that this was a better choice when dealing with larger chemical spaces, such as the ones investigated in the two case studies. All the following investigations employed the **1433** setup, where we used *Diverse Initialization Function*, *Generational Stochastic Sampling*, *Crossover Deterministic Sampling* and *Roulette Mutant Selection* allowing for duplicates.

Table S1: All the functions available in the developed EAs, along with their respective ID number used during the parametrization step.

ID	Initialization functions	Generational selection function	Crossover selection function	Mutation selection function
0	Random	Fittest	All combinations of n fittest candidates, n = 3	Fittest
1	Diverse	Roulette	All combinations of all candidates	Roulette
2		Roulette + elites = 1	Roulette	Roulette + elites = 2
3		Roulette + elites = 5	Deterministic sampling	Roulette
4		Stochastic sampling (using rank not fitness value)		Stochastic sampling
5				Stochastic sampling
6				Stochastic sampling
7				Deterministic sampling

Table S2: Ranking of the 360 different setup runs, for which each specific run has been performed 2500 times. The **Setup ID** represents the set of EA operations defined in Table S1. The efficiency percentage is the number of runs (out of a total of 2500) for which **CC3** was successfully discovered.

Setup ID	Efficiency percentage
0132	58.6
0221	58.0
0131	57.8
0233	57.6
1433	57.3
0337	57.3
1232	57.2
0231	57.1
0130	57.1
0112	57.1
1332	57.0
0332	57.0
0120	56.9
1331	56.9
0223	56.9
0333	56.8
0232	56.8
0127	56.8
0121	56.8
0113	56.7
0023	56.7
1112	56.6
0222	56.6

0322	56.5
0220	56.5
0033	56.4
1231	56.4
1131	56.4
0133	56.4
1113	56.3
0114	56.3
0022	56.2
1121	56.2
1120	56.2
0230	56.2
0134	56.1
1233	56.1
1130	56.1
0123	56.1
1221	56.0
1134	56.0
0122	56.0
0124	56.0
1321	56.0
0117	55.9
1031	55.9
0321	55.9
1122	55.8
1234	55.8

0032	55.8
0031	55.8
0331	55.7
1034	55.6
0137	55.6
1222	55.5
1132	55.4
1110	55.4
0237	55.4
0327	55.4
0224	55.4
1322	55.2
1223	55.2
1022	55.2
1320	55.2
0110	55.2
0021	55.2
1033	55.1
1023	55.1
1330	55.1
1137	55.1
0027	54.9
1337	54.9
0030	54.8
1032	54.7
1324	54.7

0234	54.6
1430	54.6
1224	54.6
1227	54.5
0320	54.5
1432	54.4
1333	54.4
1123	54.3
0422	54.3
1334	54.2
0024	54.2
1323	54.2
1230	54.2
1423	54.1
0421	54.1
1133	54.0
0227	54.0
1422	54.0
1220	54.0
1020	54.0
0311	54.0
1237	53.9
1327	53.8
0324	53.8
0334	53.7
1124	53.6

0213	53.5
0211	53.5
1027	53.4
0118	53.4
1114	53.3
1024	53.2
1311	53.1
1030	53.1
1021	53.0
1335	52.9
1037	52.9
0330	52.9
0138	52.9
0424	52.8
0338	52.6
1424	52.6
1117	52.5
1127	52.4
0235	52.1
0125	51.7
0228	51.6
1238	51.6
0135	51.6
1421	51.5
0128	51.4
1035	51.4

1235	51.4
0313	51.3
0238	51.3
1038	51.3
0325	51.2
0028	51.2
1213	51.0
0115	51.0
1211	51.0
0328	51.0
1325	51.0
1028	51.0
1212	50.9
0312	50.9
0335	50.8
1338	50.8
1435	50.7
0212	50.6
0217	50.6
1135	50.5
1125	50.5
0411	50.3
1225	50.0
1210	50.0
1025	50.0
1115	49.9

1138	49.9
0210	49.8
0317	49.6
0025	49.6
1228	49.6
1411	49.4
1313	49.4
1128	49.4
1118	49.4
0214	49.4
1310	49.2
1328	49.2
1214	49.1
1410	49.0
0435	49.0
1312	49.0
1437	48.8
1217	48.8
0413	48.4
0336	48.3
0436	48.2
1116	48.1
0310	48.0
1412	47.7
0417	47.5
1013	47.4

0314	47.4
0225	47.3
1011	47.3
0425	47.2
1136	47.2
1226	47.2
1012	47.1
0236	47.1
0010	47.1
1317	47.0
0014	47.0
0136	46.9
0012	46.9
0116	46.8
0428	46.7
1236	46.4
0036	46.4
0126	46.3
0013	46.1
1036	46.1
1314	45.9
1014	45.9
0026	45.8
1126	45.8
1336	45.6
1428	45.6

0011	45.5
0017	45.4
1326	45.3
0020	45.3
0326	45.2
0426	44.5
0226	44.5
1026	44.3
1017	43.9
0001	43.2
1010	43.1
1426	43.0
0203	42.6
0201	42.2
0103	42.2
1203	41.6
0003	41.6
1101	41.4
0303	41.4
0202	41.4
0302	41.3
1202	41.0
1003	40.9
1201	40.9
1102	40.8
1303	40.8

0107	40.8
1302	40.7
0002	40.7
0207	40.5
1436	40.4
1001	40.2
1002	40.2
1403	40.1
0301	40.0
0300	40.0
1301	39.8
1204	39.8
0407	39.8
0204	39.8
0404	39.3
0307	39.3
1103	39.2
1000	38.9
0412	38.9
0100	38.8
1104	38.7
0007	38.6
1401	38.5
1200	38.5
0215	38.5
0304	38.4

0018	38.2
1307	38.1
1318	38.1
1107	38.1
0004	38.0
1400	38.0
0015	38.0
1300	37.9
1207	37.9
0318	37.8
0000	37.8
1304	37.7
0200	37.6
1004	37.5
0218	37.5
0414	37.4
1007	37.3
1018	37.1
1404	36.9
0430	36.8
0315	36.6
1315	36.4
1015	36.3
1215	36.2
1218	35.3
0008	34.5

0005	34.4
1108	34.4
1427	34.3
0105	34.3
1105	34.2
1008	34.1
0208	34.0
1308	34.0
1208	33.9
0418	33.9
1305	33.7
0308	33.5
1005	33.2
0205	33.2
0403	32.8
1205	32.7
0305	32.7
0401	32.2
1434	31.8
0432	31.7
0433	27.5
1316	27.0
0438	26.8
1216	26.4
0415	26.4
1416	26.0

1206	25.6
0306	25.6
1420	25.4
0206	25.3
0216	25.2
0316	25.1
0006	25.1
0016	25.0
1106	25.0
1016	24.6
1006	24.4
1306	24.4
0402	22.4
1431	22.2
0434	20.4
1413	19.4
0400	18.1
0437	18.1
1407	17.3
0420	16.4
1406	15.4
1414	13.6
0423	13.0
0431	11.9
1418	11.4
0038	10.3

1405	9.7
0416	8.3
0427	7.6
0406	7.6
1438	7.3
1417	7.2
0034	6.6
1408	6.4
0405	4.8
1415	4.7
0111	4.2
0104	4.1
0106	4.1
0037	3.0
0035	2.9
1425	2.8
0102	2.0
1402	1.9
0408	1.2
0101	1.2
0410	0.7
1111	0.0
0323	0.0

CC3 rediscovery

As we optimized and calculated the fitness value for a large number of cages, it is interesting to look at the distribution of the fitness value among all the different individuals. The fitness function for this investigation employed the coefficients $a = 10, b = 10, c = 1$ from Equation 1, where we defined ideal pore and window diameters of 5.72 and 3.91 Å, respectively. In the fitness function we wanted to put a strong emphasis on the pore and window sizes in order for the global minimum to precisely match the properties of the OPLS3 optimized **CC3** cage. We also gave some importance to the level of symmetry of the cage.

Shape persistency analysis

For the shape persistency analysis, we employed the following equation:

$$\alpha = \frac{4 \times \text{window difference}}{\text{maximum diameter} \times \text{n expected windows}} \tag{3}$$

where all the properties are calculated with pyWindow and each cage has 4 windows by construction, as they all have a $\mathbf{Tri^4Di^6}$ topology. If $\alpha > 0.035$ and cavity size is greater than 1 Å, the cage is labelled as 'not collapsed', otherwise it is labelled 'collapsed'. This equation was derived through trial and error and through the visual inspection of thousands of assembled porous organic cages.

Table S3: Properties of all non-collapsed cages from the final population of run **A** for case study 1.

Rank	Pore size / Å	Window diameter / Å	Asymmetry / Å	Topology
1	5.056	3.680	0.037	$\mathbf{Tri^4Di^6}$
2	5.257	3.679	0.033	$\mathbf{Tri^4Di^6}$
3	5.067	3.798	0.068	$\mathbf{Tri^4Di^6}$
4	5.281	3.656	0.052	$\mathbf{Tri^4Di^6}$
5	5.072	3.661	0.080	$\mathbf{Tri^4Di^6}$
6	5.010	3.949	0.106	$\mathbf{Tri^4Di^6}$
7	5.192	3.721	0.082	$\mathbf{Tri^4Di^6}$
8	5.361	3.799	0.102	$\mathbf{Tri^4Di^6}$
9	5.181	3.968	0.127	$\mathbf{Tri^4Di^6}$
10	5.191	4.071	0.201	$\mathbf{Tri^4Di^6}$
11	4.563	3.222	0.215	$\mathbf{Tri^4Di^6}$
12	7.697	6.715	0.170	$\mathbf{Tri^4Di^6}$
13	5.401	3.956	0.437	$\mathbf{Tri^4Di^6}$
14	4.563	4.302	0.669	$\mathbf{Tri^4Di^6}$
15	7.655	6.590	0.433	$\mathbf{Tri^4Di^6}$
16	4.911	4.479	0.732	$\mathbf{Tri^4Di^6}$
17	2.361	2.455	0.438	$\mathbf{Tri^4Di^6}$
18	8.433	6.312	0.405	$\mathbf{Tri^4Di^6}$
19	4.538	2.328	0.719	$\mathbf{Tri^4Di^6}$
20	4.962	3.973	0.868	$\mathbf{Tri^4Di^6}$
21	8.942	7.745	0.723	$\mathbf{Tri^4Di^6}$
22	7.207	6.182	1.294	$\mathbf{Tri^4Di^6}$

Table S4: Properties of all non-collapsed cages from the final population of run **B** for case study 1.

Rank	Pore size / Å	Window diameter / Å	Asymmetry / Å	Topology
1	5.460	3.998	0.011	**Tri^4Di^6**
2	5.346	3.605	0.017	**Tri^4Di^6**
3	5.358	3.641	0.036	**Tri^4Di^6**
4	5.252	3.654	0.049	**Tri^4Di^6**
5	5.402	3.082	0.020	**Tri^4Di^6**
6	5.507	3.405	0.063	**Tri^4Di^6**
7	5.497	3.305	0.062	**Tri^4Di^6**
8	5.547	3.456	0.080	**Tri^4Di^6**
9	5.356	3.780	0.117	**Tri^4Di^6**
10	5.602	3.449	0.087	**Tri^4Di^6**
11	5.388	4.443	0.143	**Tri^4Di^6**
12	5.301	3.462	0.217	**Tri^4Di^6**
13	2.869	2.523	0.023	**Tri^4Di^6**
14	7.472	6.250	0.153	**Tri^4Di^6**
15	7.949	5.727	0.131	**Tri^4Di^6**
16	8.522	2.264	0.342	**Tri^8Di^{12}**
17	5.640	5.424	1.269	**Tri^4Di^6**
18	10.471	6.596	0.842	**Tri^8Di^{12}**
19	8.103	7.215	1.118	**Tri^4Di^6**
20	13.349	6.851	0.525	**Tri^8Di^{12}**
21	15.044	9.299	0.385	**Tri^8Di^{12}**
22	3.298	6.545	2.145	**Tri^4Di^6**
23	10.546	9.298	2.010	**Tri^4Di^6**

Table S5: Properties of all non-collapsed cages from the final population of run **C** for case study 1.

Rank	Pore size / Å	Window diameter / Å	Asymmetry / Å	Topology
1	4.960	3.862	0.067	$\mathbf{Tri^4Di^6}$
2	5.245	3.670	0.031	$\mathbf{Tri^4Di^6}$
3	5.260	3.708	0.030	$\mathbf{Tri^4Di^6}$
4	5.383	3.600	0.007	$\mathbf{Tri^4Di^6}$
5	5.362	3.629	0.045	$\mathbf{Tri^4Di^6}$
6	5.537	3.748	0.012	$\mathbf{Tri^4Di^6}$
7	4.930	4.726	0.232	$\mathbf{Tri^4Di^6}$
8	5.309	3.571	0.122	$\mathbf{Tri^4Di^6}$
9	5.482	3.261	0.077	$\mathbf{Tri^4Di^6}$
10	4.837	4.646	0.272	$\mathbf{Tri^4Di^6}$
11	4.731	3.831	0.214	$\mathbf{Tri^4Di^6}$
12	5.047	3.678	0.306	$\mathbf{Tri^4Di^6}$
13	4.998	3.728	0.435	$\mathbf{Tri^4Di^6}$
14	4.648	3.857	0.445	$\mathbf{Tri^4Di^6}$
15	4.689	3.676	0.472	$\mathbf{Tri^4Di^6}$
16	5.525	4.364	0.582	$\mathbf{Tri^4Di^6}$
17	4.691	5.270	0.826	$\mathbf{Tri^4Di^6}$
18	6.876	7.188	0.533	$\mathbf{Tri^4Di^6}$
19	8.418	2.496	0.205	$\mathbf{Tri^8Di^{12}}$
20	4.747	3.942	1.176	$\mathbf{Tri^4Di^6}$
21	5.282	6.677	1.150	$\mathbf{Tri^4Di^6}$
22	11.672	8.319	1.080	$\mathbf{Tri^4Di^6}$

Table S6: Properties of all non-collapsed cages from the final population of run **A** for case study 2.

Rank	Pore size / Å	Window diameter / Å	Asymmetry / Å	Topology
1	4.409	4.385	0.597	$\mathbf{Tri^4Di^6}$
2	4.362	5.593	0.778	$\mathbf{Tri^4Di^6}$
3	11.110	8.466	0.102	$\mathbf{Tri^4Di^6}$
4	11.384	8.451	0.187	$\mathbf{Tri^4Di^6}$
5	11.641	9.306	0.384	$\mathbf{Tri^4Di^6}$
6	12.386	10.466	0.422	$\mathbf{Tri^4Di^6}$
7	11.719	9.808	0.526	$\mathbf{Tri^4Di^6}$
8	7.753	6.186	1.094	$\mathbf{Tri^4Di^6}$
9	10.478	9.802	0.715	$\mathbf{Tri^4Di^6}$
10	12.097	10.588	0.508	$\mathbf{Tri^4Di^6}$
11	12.037	10.023	0.706	$\mathbf{Tri^4Di^6}$
12	12.568	9.779	0.690	$\mathbf{Tri^4Di^6}$
13	12.994	10.307	0.772	$\mathbf{Tri^4Di^6}$
14	11.838	10.087	0.992	$\mathbf{Tri^4Di^6}$
15	12.760	10.568	0.875	$\mathbf{Tri^4Di^6}$
16	17.119	14.706	0.277	$\mathbf{Tri^4Di^6}$
17	15.906	13.471	0.648	$\mathbf{Tri^4Di^6}$
18	15.745	13.662	1.135	$\mathbf{Tri^4Di^6}$
19	16.834	14.648	0.995	$\mathbf{Tri^4Di^6}$
20	11.423	9.543	1.940	$\mathbf{Tri^4Di^6}$
21	17.155	14.384	1.680	$\mathbf{Tri^4Di^6}$

Table S7: Properties of all non-collapsed cages from the final population of run **B** for case study 2.

Rank	Pore size / Å	Window diameter / Å	Asymmetry / Å	Topology
1	5.479	5.137	0.317	**Tri^{4}Di^6**
2	3.757	4.891	0.709	**Tri^{4}Di^6**
3	2.393	3.229	0.669	**Tri^{4}Di^6**
4	17.046	15.398	0.433	**Tri^{4}Di^6**
5	15.060	13.021	0.717	**Tri^{4}Di^6**
6	16.258	14.821	0.663	**Tri^{4}Di^6**
7	15.545	13.597	0.766	**Tri^{4}Di^6**
8	17.023	13.173	0.647	**Tri^{4}Di^6**
9	14.833	13.401	0.904	**Tri^{4}Di^6**
10	15.502	13.304	0.849	**Tri^{4}Di^6**
11	16.700	14.792	0.762	**Tri^{4}Di^6**
12	16.428	14.326	0.850	**Tri^{4}Di^6**
13	17.515	14.391	0.800	**Tri^{4}Di^6**
14	17.660	14.848	0.821	**Tri^{4}Di^6**
15	20.759	18.392	0.643	**Tri^{4}Di^6**
16	15.674	13.112	1.465	**Tri^{4}Di^6**
17	20.362	19.202	0.913	**Tri^{4}Di^6**
18	16.076	15.910	1.430	**Tri^{4}Di^6**
19	15.707	13.489	1.605	**Tri^{4}Di^6**
20	14.518	17.499	2.164	**Tri^{4}Di^6**
21	19.732	18.137	2.014	**Tri^{4}Di^6**

Table S8: Properties of all non-collapsed cages from the final population of run **C** for case study 2.

Rank	Pore size / Å	Window diameter / Å	Asymmetry / Å	Topology
1	12.046	8.309	0.006	$\mathbf{Tri^4Di^6}$
2	11.955	9.286	0.242	$\mathbf{Tri^4Di^6}$
3	11.752	9.786	0.425	$\mathbf{Tri^4Di^6}$
4	8.347	7.741	0.912	$\mathbf{Tri^4Di^6}$
5	15.623	12.446	0.253	$\mathbf{Tri^4Di^6}$
6	16.430	13.251	0.218	$\mathbf{Tri^4Di^6}$
7	12.289	9.746	0.633	$\mathbf{Tri^4Di^6}$
8	16.554	13.534	0.293	$\mathbf{Tri^4Di^6}$
9	12.179	9.857	0.747	$\mathbf{Tri^4Di^6}$
10	16.161	13.153	0.356	$\mathbf{Tri^4Di^6}$
11	16.437	13.144	0.356	$\mathbf{Tri^4Di^6}$
12	16.762	13.444	0.351	$\mathbf{Tri^4Di^6}$
13	15.338	12.543	0.500	$\mathbf{Tri^4Di^6}$
14	16.505	13.161	0.406	$\mathbf{Tri^4Di^6}$
15	16.748	14.550	0.377	$\mathbf{Tri^4Di^6}$
16	16.550	13.761	0.411	$\mathbf{Tri^4Di^6}$
17	15.135	12.597	0.564	$\mathbf{Tri^4Di^6}$
18	11.740	9.355	1.177	$\mathbf{Tri^4Di^6}$
19	7.238	8.133	1.605	$\mathbf{Tri^4Di^6}$
20	11.510	9.703	1.264	$\mathbf{Tri^4Di^6}$
21	10.045	10.910	1.894	$\mathbf{Tri^4Di^6}$
22	10.869	9.525	2.203	$\mathbf{Tri^4Di^6}$

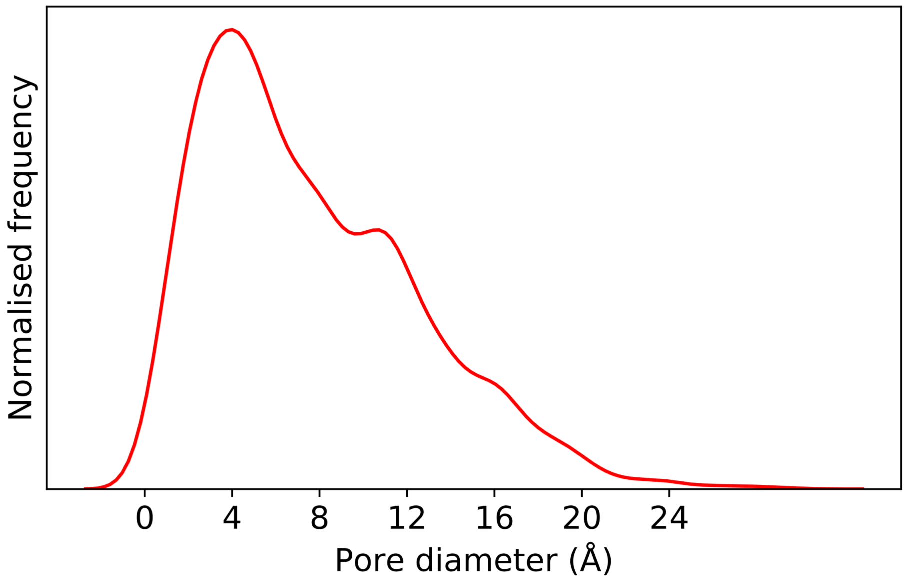

Figure S1: Distribution of pore diameters for the 5772 shape persistent porous organic cages generated within the mock chemical space defined in the **CC3** rediscovery section.
